# Exercise testing in the assessment of patients with bronchiectasis

**DOI:** 10.36416/1806-3756/e20260267

**Published:** 2026-08-13

**Authors:** Danilo C Berton, Carla Tatiana Martins de Oliveira, Denis E O’Donnell, José Alberto Neder

**Affiliations:** 1. Unidade de Fisiologia Pulmonar, Hospital de Clínicas de Porto Alegre, Programa de Pós-Graduação em Ciências Pneumológicas, Universidade Federal do Rio Grande do Sul, Porto Alegre (RS) Brasil.; 2. Pulmonary Function Laboratory and Respiratory Investigation Unit, Division of Respirology, Kingston Health Science Center & Queen’s University, Kingston (ON) Canada.

## BACKGROUND

In the last two issues of this series, the role of resting pulmonary function tests in the assessment and management of patients with cystic fibrosis (CF) and non-CF bronchiectasis was extensively reviewed. Resting functional measurements, however, correlate variably with the functional capacity of patients and do not accurately reveal the mechanism(s) of exercise intolerance or accurately predict the prognosis in patients with chronic cardiopulmonary conditions.^1^

## OVERVIEW

**Figure 1:**
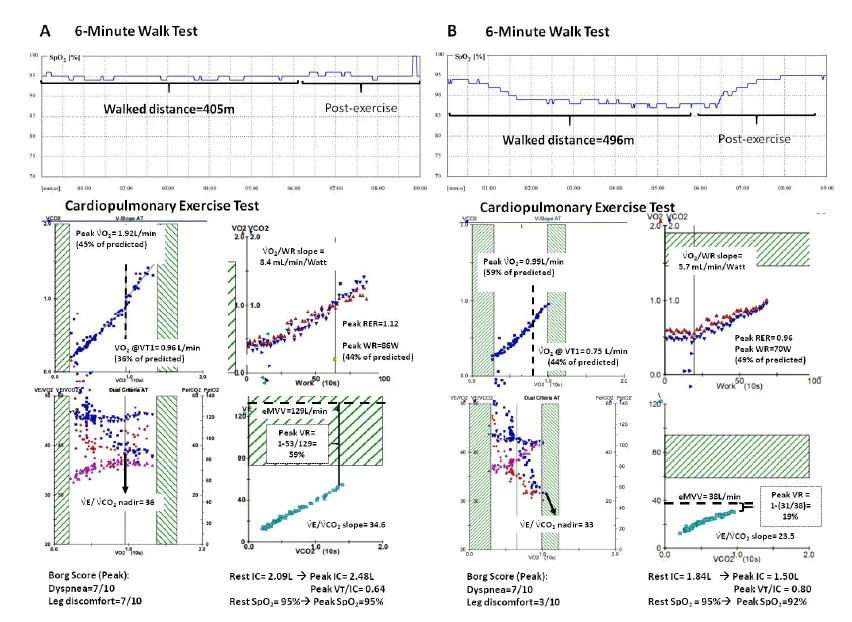
In A, a 40-year-old male patient with cystic fibrosis (CF) had mild obstructive ventilatory disorder (FEV_1_ = −1.86 z-score) and a six-minute walk distance (6MWD) > 400 m, without exercise-induced desaturation (EID). Cardiopulmonary exercise testing (CPET) on a cycle ergometer showed severely reduced aerobic capacity and exercise tolerance. Low V̇O_2_ at ventilatory threshold 1 (VT1 = lactate threshold) and a reduced V̇O_2_/work rate (WR) slope, with one objective criterion for maximum effort (peak respiratory exchange ratio [RER] = 1.12), indicated that oxygen delivery/utilization dysfunction was the predominant mechanism of exercise intolerance. Notably, there was no evidence of ventilatory limitation during exercise, with peak ventilatory reserve (VR) within normal limits and increasing inspiratory capacity (IC) without reaching a critically high V_T_/IC ratio at peak effort (< 0.70). In B, a 46-year-old male patient with CF and severe obstructive ventilatory disorder (FEV_1_ = −6.63 z-score) had a 6MWD of 496 m, with EID. Cycling CPET showed low aerobic capacity and exercise intolerance. Exercise was interrupted with dyspnea greater than leg discomfort and an RER = 0.96, although other markers indicated a maximal test with reduced peak VR, decreasing IC, and a critical V_T_/IC ratio at peak effort (> 0.70). The progressive decrease in ventilatory equivalents without compensatory increase after VT1, associated with a progressive increase in PETCO_2_ and a progressive decrease in PETO_2_ until exercise interruption, reinforces the impression of ventilatory limitation during exercise. V̇O_2_ at the lower limit of normal at VT1 and a reduced V̇O_2_/WR slope also indicate a component of oxygen delivery/utilization dysfunction. Of note, the intensity of desaturation during exercise is greater when walking during the six-minute walk test (6MWT) than when cycling during CPTE. Abbreviations: PetCO_2_ = end-tidal carbon dioxide pressure; PetO_2_ = end-tidal oxygen pressure;V̇CO_2_: carbon dioxide production; and eMVV: estimated maximal voluntary ventilation.

A 40-year-old male patient with CF complained of exercise intolerance despite only a mild obstructive ventilatory defect (FEV_1_ = −1.86 z-score). He walked 405 m during the six-minute walk test, without exercise-induced desaturation. Cardiopulmonary exercise testing on a cycle ergometer revealed low aerobic capacity, with similarly high dyspnea and leg discomfort (Borg scale scores = 7/10) despite a reduced peak work rate (WR; 86 W). There was no evidence of exercise-induced ventilatory limitation, as observed by a peak ventilatory reserve within normal limits and an increment of inspiratory capacity (IC) without reaching a critically high peak V_T_/IC ratio (< 0.70). Low V̇O_2_ at ventilatory threshold 1 and a shallow V̇O_2_/WR slope indicated that impaired oxygen delivery/utilization was the predominant mechanism of exercise intolerance (Figure 1A).^2^ A 46-year-old male patient with CF and a more severe obstructive ventilatory defect (FEV_1_ = −6.63 z-score) interrupted the cardiopulmonary exercise test with dyspnea greater than leg discomfort, a respiratory exchange ratio = 0.96, reduced peak ventilatory reserve, decreasing IC, and a critically high V_T_/IC ratio at peak effort (= 0.80). Note that a respiratory exchange ratio of < 1.05 is a common finding in ventilatory-limited patients, and, in this instance, it does not necessarily indicate submaximal exercise. Beyond exertional ventilatory limitation, V̇O_2_ at the lower limit of normal at ventilatory threshold 1 (44% of predicted) and a reduction in the V̇O_2_/WR slope also indicate a component of oxygen delivery/utilization dysfunction (Figure 1B).^2^

Most evidence on the mechanisms of exercise intolerance and the role of exercise impairment in prognosis assessment comes from bronchiectasis patients with CF; non-CF patients are usually older and may present with associated cardiopulmonary comorbidities, and this creates challenges in teasing out the contribution of the respiratory system to the shortness of breath of patients. Patients with mild CF appear to have preserved exertional ventilatory response and are commonly limited by nonrespiratory factors, such as central and/or peripheral mechanisms that alter oxygen transport and utilization (patient 1, Figure 1A). As respiratory impairment progresses (i.e., with a reduction in FEV_1_), the mechanisms underlying reduced exercise performance and increased dyspnea perception are mainly ventilatory (patient 2, Figure 1B).^2^ Objective exercise testing demonstrated worse performance in patient 1, despite he having a less severe FEV_1_ impairment than patient 2. Conversely, although patient 2 faced advanced resting and exertional ventilatory limitation, oxygen delivery/utilization dysfunction also played a relevant role, both patients therefore being excellent candidates for pulmonary rehabilitation. 

## CLINICAL MESSAGE

Clinical exercise tests provide an objective way to measure exercise capacity and reveal the potential mechanism(s) of exercise intolerance. Cardiopulmonary exercise testing and field walking tests are useful prognostic tools, providing predictive indicators of relevant clinical outcomes such as death and lung transplantation. They have been demonstrated to be reliable and reproducible assessments of exercise capacity, being responsive to intervention in patients with bronchiectasis.^1^ A summary of the clinical utility of clinical exercise tests is provided in the figure footnote. 

## CLINICAL EXERCISE TESTS PECULIARITIES IN PATIENTS WITH BRONCHIECTASIS

- CF patients with the highest peak V̇O_2_ (> 82% of predicted) or in the intermediate category (59-81% of predicted) had, respectively, a 72% (hazard ratio = 0.278; 95% CI, 0.088-0.882) and 49% (hazard ratio = 0.507; 95% CI, 0.259-0.993) lower risk of dying or receiving a lung transplant in the following 10 years when compared with patients in the lowest peak V̇O_2_ category (< 58% of predicted).^3^
- Higher V̇E/V̇CO_2_ (nadir, slope, and intercept) is a clear feature of adults with CF, even among those with normal spirometry. Apparently free from interference from mechanical constraints, V̇E/V̇CO_2_ nadir appears to be the most reliable parameter to evaluate excess exercise ventilation in adults with CF,^4^ given that the V̇E/V̇CO_2_ slope may even decrease as obstructive airway disease worsens.^5^
- The minimum clinically important difference (i.e., the smallest change in an outcome recognized as beneficial by the patient and that would lead to a change in treatment) in individuals with bronchiectasis was estimated at 25 m for the 6MWT and 35 m for the incremental shuttle walk test.
- There is a greater drop in SpO_2_ during field walking than during cycling CPET.
- For every 10 m walked during the 6MWT, there is a 6% decrease in the risk of death.
- A 6MWD < 400 m or EID is a criterion for referring adults with CF with an FEV_1_ of < 40% predicted or children with CF with an FEV_1_ of < 50% predicted for lung transplantation. Similar criteria are applied to non-CF bronchiectasis, although many patients have a more stable condition.^6^
